# Bayesian prevalence of autism and unmet special education needs in Chile in a sample of three million school-age children

**DOI:** 10.1177/13623613251342310

**Published:** 2025-08-04

**Authors:** Andres Roman-Urrestarazu, Adele Tyson, Gabriel Gatica-Bahamonde, Robin van Kessel, Justin Yang, Carola Mansilla, Isabel Zuniga, Alejandra Méndez-Fadol, Blanca Larrain, Ricardo Garcia, Damaris Koch, Tamsin Ford, Wim Groot, Milena Pavlova, Katarzyna Czabanowska

**Affiliations:** 1 Department of Psychiatry, University of Cambridge, UK; 2 Department of International Health, Faculty of Health, Medicine and Life Sciences, Maastricht University, The Netherlands; 3 Seccion de Psiquiatria del Niño y el Adolescente, Facultad de Medicina, Pontificia Universidad Católica de Chile, Chile; 4 LSE Health, Department of Health Policy, London School of Economics and Political Science, UK; 5 Division of Psychiatry, University College London, UK; 6 Fundación Mis Talentos, Chile; 7 Departamento de Pediatria y Cirugía Infantil, Facultad de Medicina, Universidad de la Frontera, Chile; 8 Dpto. de Psiquiatría y Salud Mental de la Infancia y la Adolescencia. Facultad de Medicina, Universidad de Chile, Chile; 9 Cambridgeshire and Peterborough NHS Foundation Trust, UK; 10 Department of Health Policy Management, Institute of Public Health, Faculty of Health Sciences, Jagiellonian University, Krakow, Poland

**Keywords:** ASD prevalence, Bayesian methods, Chile, Latin America, special education needs

## Abstract

Autism spectrum disorder prevalence estimates in Latin America have been limited by a lack of reliable data. This cohort study aimed to estimate autism spectrum disorder prevalence in Chile by linking school registries with electronic health records. Probabilistic data linking was conducted between the 2021 Chilean school registry and 2003−2015 health records from the Araucania Sur Health Service. Bayesian prevalence estimation was used, considering variables such as age, sex, immigration status, ethnicity and rurality. Autism spectrum disorder prevalence across 29 health services was analysed for students aged 6 to 18 years. Regression models assessed unmet needs for special education services, validated using Araucania Sur Health Service health records. The study included 3,056,306 pupils (51.34% boys; mean age 11.6 years), with 14,549 having autism spectrum disorder. The national standardized school prevalence was 0.46%. Boys had 6 times higher odds of receiving special education services support than girls. In Araucania Sur Health Service, the adjusted clinical prevalence from health records was 1.22%. The Bayesian projected national autism spectrum disorder prevalence was 1.31%. This study, the largest of its kind in Latin America, revealed a higher autism spectrum disorder prevalence than previously reported, with one in 76 children affected. Disparities were observed across sex, ethnicity and health services.

**Lay abstract:**

This project tried to understand how many children in Chile are affected by autism, as reliable data have been lacking not only in Chile but across much of Latin America. To do this, we carried out the largest autism prevalence study ever conducted in the region. We linked national school records from 2021 with over a decade of health records (2003–2015) from the Araucanía Sur Health Service in southern Chile. This allowed us to examine data from more than three million students aged 6 to 18 years across 29 health services. Our results revealed that around one in every 76 children may have autism – almost 3 times higher than what was reported in schools alone. We also found that boys were 6 times more likely than girls to receive special education support. Using advanced statistical modelling, we estimated a national autism prevalence rate of 1.31%. Importantly, we discovered disparities in diagnosis and access to support based on sex, ethnicity, immigration status and whether a child lived in a rural or urban area. These findings highlight the need for more inclusive and equitable approaches to autism identification and care across Chile. This research not only helps to fill a major data gap but also offers a model for how countries with limited resources can use existing administrative data to improve public health planning and educational support for children with autism.

## Introduction

Autism spectrum disorder (ASD) is a neurodevelopmental condition that affects social interaction and communication and affects between 1% and 2% of the global population (Roman-Urrestarazu et al., 2021, 2022). Recently, there has been a growing interest in understanding ASD prevalence, particularly using large research designs such as school registries and access to special education needs (SEN) services from school registry data (Roman-Urrestarazu et al., 2021, 2022), but little is known how this relates to clinical prevalence, and few studies have linked national registries to electronic health records (Patrick et al., 2023) to study the gap between those diagnosed but not receiving support at school (Elsabbagh et al., 2012; Roman-Urrestarazu et al., 2020; Zeidan et al., 2022). One of the challenges when studying the prevalence of ASD in Latin America and the Caribbean is the lack of reliable data sources, which is reinforced by the fact that only 2.3% of the prevalence studies published worldwide to date are based in this region, as well as by the fact that to date there are few prevalence studies in Latin America (Montiel-Nava et al., 2017; Paula et al., 2014). Chile is a high-income country with a population of 19 million people (Instituto Nacional de Estadistica, 2017) and ranks as one of the wealthiest and most unequal Latin American countries (World Bank, 2023), with significant disparities in health outcomes according to socioeconomic status (SES; Frank & Matsunaga, 2020; Organisation for Economic Co-Operation and Development [OECD], 2018). Such inequities in access, higher unmet need for those unable to pay, and longer waiting times for those using non-private providers affect autistic people (Pan American Health Organization [PAHO], 2015; Roman-Urrestarazu et al., 2020). To address inequalities in ASD diagnosis and screening, Chile legislated a new ASD law in 2023 (Guajardo Sáez, 2021), which establishes inclusion, comprehensive care and protection of the rights of people with ASD, although accurate epidemiological estimates are needed for this law to have a meaningful effect.

As such, we aimed to examine the prevalence of ASD in Chilean school-aged children aged between 6 and 18 years using a mix of registry data from the Chilean school SEN inclusion programme (*Programa de Integracion Escolar; PIE*) and electronic health records in one of Chile’s 29 regional health services. Specifically, we first report the prevalence of autistic children in the Chilean PIE. Second, we assess access determinants to ASD SEN services in Chile using a two-level mixed-effects logistic regression model. Third, we validated school-level prevalence estimates with a probabilistic linkage of clinical subset of electronic health records of autistic children in one of Chile’s 29 regional health service to the school registry since not all children with recognized ASD participate in SEN programmes, which allowed us to estimate the unmet need for SEN services between school-level services and clinical diagnosis. Finally, we developed a Bayesian prevalence model to extrapolate nationally our prevalence estimates from our subsample analysis across Chile’s 28 other health services, seeking to identify disparities in ASD prevalence and access to SEN services. This study has important implications for policy and resource allocation related to the education and support of children with ASD in Chile and Latin America and the Caribbean.

## Methods

### The Chilean PIE school registry and electronic health record data

**Figure 1.**
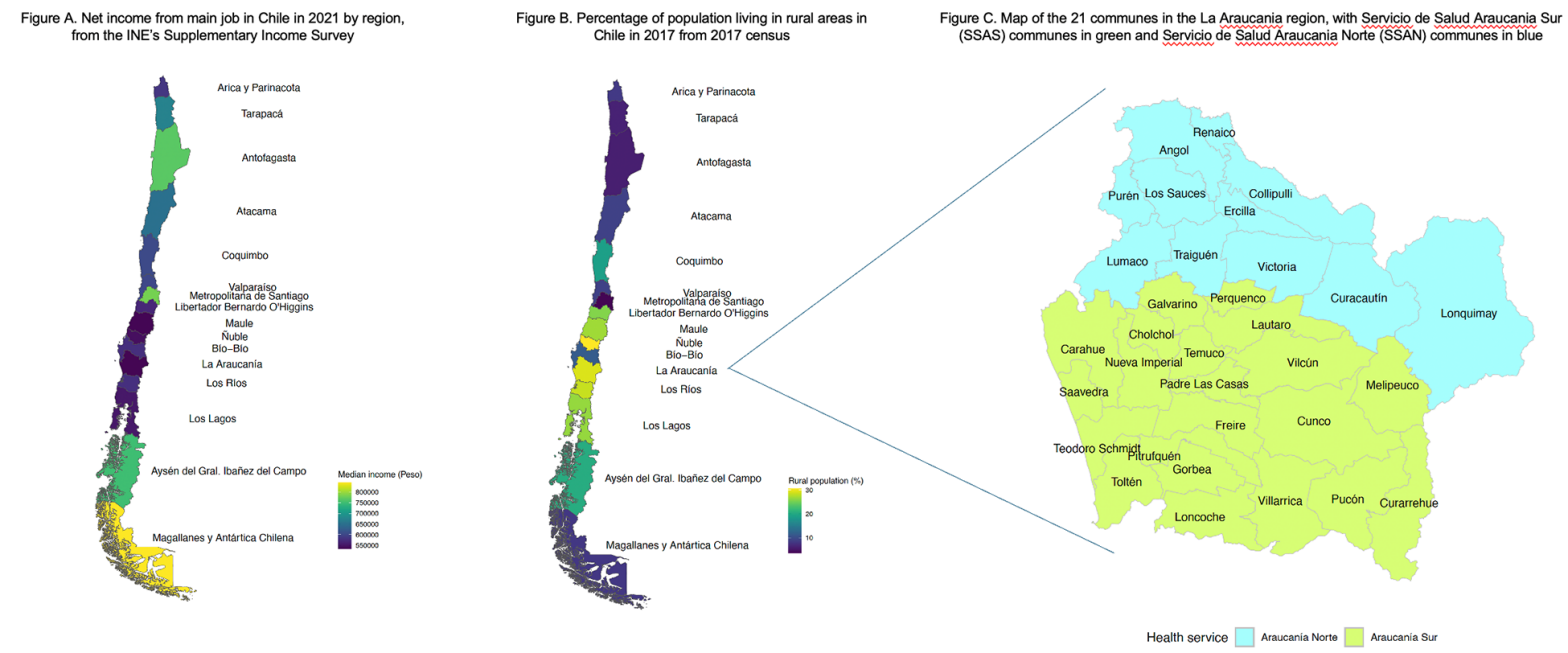
Map of Chile’s regions and rurality with zoom into the Araucania region.

The 2021 Chilean school registry covers the total student population in Chile together with schools that participate in the PIE programme, and is collected by the Ministry of Education (eMethods 1). This study followed the Strengthening the Reporting of Observational Studies in Epidemiology (STROBE) reporting guideline and was approved by the corresponding Ethics Committees. Anonymized data access was granted by the Ministry of Education in May 2023, and anonymized health care contacts between 2014 (year of introduction) and 2021 were obtained from the electronic health records from the *Servicio de Salud Araucania Sur* (SSAS) health service that covers 21 municipalities in the province of Cautín, Araucanía region (IX), through primary, secondary and tertiary health care providers and includes a regional referral hospital together with an intercultural hospital that service the Mapuche people who are Chile’s main native American group. The data sets generated and analysed during this study are not publicly available due to our agreement with the public institutions that provided the data. Our agreement with them does not include permission to share the data. The sample is representative of the general Chilean population by including a large metropolitan centre (Temuco), large rural areas and an oversampling of Chile’s main indigenous group the Mapuche people. Data were collated from secondary care records and mental health community services and comprise health visits of patients aged 6 to 18 years with a primary diagnosis of ASD for all communes in the SSAS catchment area. Two datasets were derived from this, which will be referred to as the (1) clinical dataset, and a subset of this, namely, the (2) clinical validation subsample. The clinical validation subsample was taken from Villarrica Hospital catchment area, the SSAS second-largest facility, to evaluate the accuracy of ASD diagnoses (see Figure 1 and eMethods 2).

### Operationalizing ASD status from school registry data

The PIE is a school-specific learning programme and has 28 different categories of provision, which are divided into permanent and transitory categories (eMethods 3). Diagnosis is made by child and adolescent psychiatrists or paediatric neurologists registered in Chile’s health services regulator (*Superintendencia de Salud*). The Chilean school registry shows whether students have accessed the PIE through the Differential Special Education Grant (*Subvención de Educación Especial Diferencial-SEED*), and whether they require adjustments such as specialist schools or small class sizes. The Chilean school registry includes only one SEN category per pupil binarily coded, based on whether a child meets the diagnostic criteria for ASD (codes F84.0 to F84.9) from the *International Statistical Classification of Diseases and Related Health Problems, Tenth and Eleventh Revision* (*ICD-10* and *ICD-11*).

### Independent variables and regional analysis units in school registry

In the school registry dataset, we coded age in 3-year bands (primary school: 6−8, 9−11; secondary school: 12−14 and 15−18 years), sex (binarily assigned at birth), immigration status (yes or no), monthly school fees converted to U.S. dollars (free, US$1.15−US$11.50, US$11.51−US$28.75, US$28.76−US$57.51, US$57.52−US$115.01, >US$115.02, missing), ethnicity (Mapuche, Aymara, Other, and No native groups) and rurality (yes or no) as our independent variables. Chile has 15 regions, which are serviced by 29 health services. Pupils’ address was mapped to their respective health service catchment area. Missing data on address were imputed using their school’s commune. We report ASD school prevalence across Chile’s 29 health services as a measure of access to SEN services. We then selected the SSAS as our clinical validation catchment area, which covers 21 communes in the province of Cautín, Araucanía region (IX) through primary, secondary, and tertiary health care providers (eMethods 4). Its large catchment area (*N* = 752,100) represents 4.3% of the total Chilean population (*N* = 17,574,003) (Instituto Nacional de Estadistica, 2017).

### Linking school registry and clinical data

To assess the unmet need for ASD in the school registry, its relationship to clinical diagnoses, and to provide corrected national estimates, we used a clinical validation sample. To show the validity of the clinical diagnoses taken from the electronic health records, a subset of this clinical sample was manually reviewed by both a child and adolescent psychiatrist and a paediatric neurologist who participate in the PIE diagnostic process and the interrater agreement was computed using Cohen’s Kappa (Cohen, 1960). If a child’s record indicated that the ASD case definition had been met, they then obtained information from the child’s developmental evaluations, PIE plans, and other documents (e.g. cognitive or IQ tests) and combined records across data sources. Children met the ASD case definition if they were between 6 and 18 years old in 2021, lived in the SSAS Health Service catchment area during 2021, and had ever received any of the following: a written statement from a qualified professional diagnosing ASD, a PIE classification of ASD, or were given an *ICD-10* code from F84.0 to F84.9. The remaining records were then matched to the Chilean national school registry, using Fellegi−Sunter probabilistic data linkage (Fellegi & Sunter, 1969), based on date of birth, sex, a SES proxy based on insurance status and school fees, and commune of residence. We performed clerical review of machine-generated matched pairs based on the calculated field-similarity scores for each input field individually, and then used a composite pair-similarity score (eMethods 5). The candidate with the highest pair-similarity score was chosen as a match.

## Statistical analyses

### Frequentist analysis of school registry data and determinants of access to ASD SEN

Data were analysed from 1 May 2023 to 25 October 2023 in R version 4.3.1 (R Team, 2010). Raw national prevalence estimates for ASD in Chilean schools were directly standardized and stratified by age and sex, using the Chilean 2017 census projections for 2021 as a standard population to calculate national prevalence across 29 health services (Roman-Urrestarazu et al., 2021). To assess determinants to ASD SEN access, adjusted relative risk (referred to henceforth as adjusted prevalence ratio [aPR]) estimates were obtained using a Poisson regression with robust error variance using pupils with ASD SEN as our outcome variable (Roman-Urrestarazu et al., 2021). In each outcome model, we used the same independent variables of sex, age band, immigration status, ethnic group, school fees and rurality and included them in the same adjusted model comparing all levels against each other and reporting for missing data. Gamma confidence limits were calculated at the 95% level using chi-square distributions. To control for multiple comparisons, we used a significance level of two-sided p < 0 .001 for all reported outcomes. We then conducted a sensitivity analysis comparing the Poisson regression model with a two-level mixed-effects logistic regression model with two random intercepts at the school and commune level to calculate odds ratios (ORs) for ASD SEN access in Chilean schools, adjusting for the same independent variables as in the Poisson model, and comparing model fit.

### Bayesian prevalence estimates using school, clinical, and linked data and unmet need for SEN

After calculating the prevalence of ASD using school data, we used the linked data to calculate clinical ASD prevalence in the SSAS. The adjusted prevalence delta was calculated as the ratio difference between the adjusted prevalence of the SSAS linked data and school registry data. For the purpose of this analysis, we assumed this ratio was applicable nationally. This ratio was then extrapolated to the 28 health services other than SSAS by using it as prior in a Bayesian random-effects model on health service to calculate the adjusted prevalence projections. Estimated credible intervals were calculated for the projections by finding the maximum band around the projection of equal width to the 95% gamma confidence interval for each health service’s school data−adjusted prevalence. Bayesian prevalence analysis of ASD allowed us to calculate ASD prevalence inference with different types of incomplete data allowing plausible national prevalence estimates and providing information about the likelihood of these predictions given the observed school data (for methods and model used, see eMethods 7). Bayesian prevalence modelling was implemented in the Just Another Gibbs Sampler (JAGS) language, which uses Markov chain Monte Carlo (MCMC) sampling to produce posterior density distributions when given the above priors and adjusted prevalence observations (van de Schoot et al., 2021). A burn-in period of 2000 samples was used to ensure models converge, and then 2000 iterations without thinning were used to model the posterior densities checking for convergence (Plummer et al., 2016).

### Community statement

Our authors include neurodiverse individuals, groups from the Chilean autism community, family members, together with community organizations. These people were involved in the development of the research question, study design, data collection, implementation, analysis, interpretation or dissemination of the findings.

## Results

### Descriptive statistics and frequentist prevalence estimation using school data

Our final school dataset consisted of 3,056,306 children aged 6 to 18 years (boys *N* = 1,569,082; 51.34%) (Table 1). A PIE code was recorded for *N* = 339,968 children (11.12%), and 48.41% of 12,077 Chilean schools were participating in the PIE programme. A total of 14,549 pupils with ASD were identified in the school registry (boys *N* = 12,571 (86.40%), girls *N* = 1,978 (13.60%)). The adjusted prevalence of ASD in our national school sample was 0.46% (95% confidence interval (CI) = [0.45%, 0.47%]), with a prevalence in boys of 0.79% (95% CI = [0.77%, 0.80%]) and in girls of 0.13% (95% CI = [0.13%, 0.14%]), for a male to female ratio (MFR) ratio of 6:1.

**Table 1.** Count and percentage of features’ values in the school dataset.

	Count (%)
Sex	
Girls	1,487,224 (48.66%)
Boys	1,569,082 (51.34%)
Age band	
6−8	748,406 (24.49%)
9−11	767,350 (25.11%)
12−14	749,693 (24.53%)
15−18	790,857 (25.88%)
Health service	
Aconcagua	46,840 (1.53%)
Aisén	19,890 (0.65%)
Antofagasta	119,378 (3.91%)
Araucanía Norte	36,651 (1.20%)
Araucanía Sur	132,242 (4.33%)
Arauco	31,318 (1.02%)
Arica	44,609 (1.46%)
Atacama	58,743 (1.92%)
Biobío	71,411 (2.34%)
Chiloé	30,908 (1.01%)
Concepción	109,502 (3.58%)
Coquimbo	141,152 (4.62%)
Iquique	69,935 (2.29%)
Magallanes	28,031 (0.92%)
Maule	182,352 (5.97%)
Metropolitano Central	122,576 (4.01%)
Metropolitano Norte	180,230 (5.90%)
Metropolitano Occidente	277,282 (9.07%)
Metropolitano Oriente	182,798 (5.98%)
Metropolitano Sur	200,984 (6.58%)
Metropolitano Sur Oriente	236,817 (7.75%)
O’Higgins	161,335 (5.28%)
Osorno	40,266 (1.32%)
Reloncaví	79,767 (2.61%)
Talcahuano	54,678 (1.79%)
Valdivia	66,206 (2.17%)
Valparaíso	78,598 (2.57%)
Viña del Mar	172,456 (5.64%)
Ñuble	79,351 (2.60%)
School fee	
Free	2,190,359 (71.67%)
US$1.15 to US$11.50	1,120 (0.04%)
US$11.51 to US$28.75	36,477 (1.19%)
US$11.51 to US$28.75	206,952 (6.77%)
US$57.52 to US$115.01	270,875 (8.86%)
>US$115.02	300,521 (9.83%)
Missing	50,002 (1.64%)
Ethnicity	
Mapuche	176,302 (5.77%)
Aymara	20,946 (0.69%)
Other native group	21,692 (0.71%)
No native group	2,837,366 (92.84%)
Rurality	
Rural	238,948 (7.82%)
Urban	2,817,358 (92.18%)
Accesses SEN	
Yes	339,968 (11.12%)
No	2,716,338 (88.88%)
ASD	
Yes	14,549 (0.48%)
No	3,041,757 (99.52%)

There were *N* = 176,302 (5.77%) Mapuche and *N* = 20,946 (0.69%) Aymara pupils in our sample and 21,692 students declared to belong to other indigenous groups. We found an adjusted ASD school prevalence of 0.35% (0.32%−0.38%) for Mapuche pupils, with 0.07% (95% CI = [0.05%, 0.09%]) Mapuche girls and 0.62% (95% CI = [0.57%, 0.67%]) Mapuche boys (MFR: 8.86:1) being autistic, and 0.65% (95% CI = [0.55%, 0.80%]) for Aymara pupils with a prevalence of 0.17% (95% CI = [0.17%, 0.24%]) for Aymara girls and 1.15% for Aymara boys (MFR: 6.8:1), and 0.47% for all other indigenous groups (95% CI = [0.39%, 0.63%]), with a prevalence for girls of 0.12% (95% CI = [0.05%, 0.19%]) and for boys of 0.81% (95% CI = [0.63%, 0.98%]) for all other ethnic indigenous groups (MFR: 6.75:1). These rates are lower and higher, respectively, than those not declaring to belong to this groups: 0.47% (95% CI = [0.46%, 0.48%]) with an ASD prevalence of 0.13% (95% CI = [0.13%, 0.14%]) for girls and 0.79% (95% CI = [0.78%, 0.81%]) for boys, which contrast to the MFR in non indigenous children of MFR: 6.1:1. Immigrant children reported an adjusted ASD prevalence of 0.19% (95% CI = [0.17%, 0.21%]).

ASD prevalence varied by health service (Table 3) with the highest adjusted prevalence being reported in Ñuble (1.29%, 95% CI = [1.21%, 1.37%), and the lowest in Metropolitano Norte (0.29%, 95% CI = [0.26%, 0.31%]). It is also low in the Metropolitano health services which serve Santiago, Chile’s largest city, all had a prevalence below 0.40%. ASD peaked in the 6 to 8 age band across all services except Chiloé and Magallanes where it peaked in the 9 to 11 band. The adjusted prevalence in Chilean schools was considered the lower bound on the true prevalence of ASD in Chile for our Bayesian prevalence model.

### Analysis of access to ASD SEN in schools

Our two-level mixed effect logistic regression model shows similar results with boys having 6 times higher odds to have a diagnosis and to receive SEN support than girls (OR = 6.10, 95% CI = [5.82, 6.41]). Compared with children aged 5 to 8 years, all other age groups showed lower odds of ASD, as did immigrants, and Mapuche children. Compared with children who pay no school fees, all children in school fee categories of US$10,001 or higher reported lower odds of ASD. Children from rural areas reported higher odds of ASD compared with children from urban regions (1.19, 95% CI = [1.10, 1.30]). Our likelihood-ratio test asserts that our two-level mixed-effects model was an improvement over a simple Poisson regression mode (χ^2^ = 9112.46; p < 0.001). Further details are also shown in Table 2.

**Table 2.** Poisson and hierarchical regression estimates for SEN Access. The hierarchical model is nested at the school and commune levels.

Variable	Level	Poisson model		Hierarchical model	
		Adjusted prevalence ratio (95% CI)	p-value	Odds ratio (95% CI)	p-value
Sex	Girls	Reference		Reference	
	Boys	6.01 (5.73−6.30)	<0.001	6.10 (5.82−6.41)	<0.001
Age category	5−8 years	Reference		Reference	
	9−11 years	0.79 (0.76−0.82)	<0.001	0.77 (0.74−0.81)	<0.001
	12−14 years	0.58 (0.56−0.61)	<0.001	0.60 (0.57−0.62)	<0.001
	15−18 years	0.39 (0.37−0.41)	<0.001	0.44 (0.41−0.46)	<0.001
Immigration status	Non-immigrant	Reference		Reference	
	Immigrant	0.34 (0.31−0.38)	<0.001	0.26 (0.23−0.29)	<0.001
Ethnicity	Non-native	Reference		Reference	
	Aymara	1.21 (1.03−1.43)	0.021	1.17 (0.97−1.41)	0.104
	Mapuche	0.61 (0.56−0.66)	<0.001	0.71 (0.65−0.77)	<0.001
	Other	0.92 (0.77−1.11)	0.397	0.98 (0.81−1.20)	0.870
School fees	None	Reference		Reference	
	US$1.15−US$11.50	1.15 (0.58−2.29)	0.688	0.68 (0.20−2.31)	0.535
	US$11.51−US$28.75	0.36 (0.29−0.45)	<0.001	0.29 (0.20−0.42)	<0.001
	US$11.51−US$28.75	0.55 (0.50−0.59)	<0.001	0.52 (0.45−0.59)	<0.001
	US$57.52−US$115.01	0.67 (0.63−0.72)	<0.001	0.56 (0.50−0.63)	<0.001
	>US$115.02	0.08 (0.07−0.09)	<0.001	0.04 (0.03−0.05)	<0.001
	Missing	0.81 (0.71−0.91)	0.001	0.52 (0.41−0.64)	<0.001
Rurality	Urban	Reference		Reference	
	Rural	1.07 (1.01−1.13)	0.014	1.19 (1.10−1.30)	<0.001

### Probabilistic data linkage, unmet need for SEN, and prevalence from the clinical validation sample

**Figure 2.**
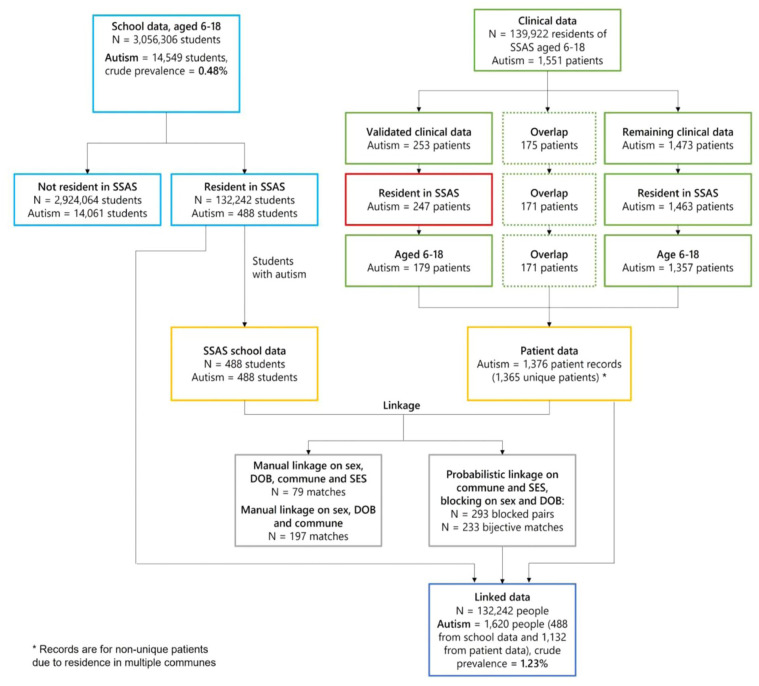
Data flow of school and clinical validation sample.

After blocking on sex and date of birth, we obtained 293 blocked pairs (Figure 2). Probabilistic matching performed using sex, date of birth, commune of residence and the proxies for SES with selection of possible matches to create a bijective set of matches resulted in 233 matches of unique SSAS school and patient records. Cohen’s Kappa for interrater reliability on the diagnostic validation subsample was 0.97, which indicates excellent agreement. This corresponds to 47.65% of the school records for students with ASD in SSAS having a match in the SSAS patient records, 16.93% of the patient records having a match in the SSAS school records and 17.07% of the unique patients having a match in the SSAS school records. For each patient who had lived in more than one commune and therefore appeared more than once in the patient data, only one match to an SSAS school record was made, meaning the matching was bijective for SSAS school records and unique patients. After linking the school and patient data for SSAS, 1132 patients with ASD could not be matched to students in the school registry. This represents the unmet need of SSAS students with ASD who did not access school-based support for it. Combining these additional cases with the 488 students who did access SEED for ASD gave 1620 people with ASD in SSAS that has a population of *N* = 132,242 children aged 6 to 18 years. The crude updated prevalence of ASD in SSAS was 1.23% (95% CI = [1.17%, 1.28%]) and the adjusted updated prevalence of ASD was 1.22% (95% CI = [1.16%, 1.28%]). For girls, the adjusted SSAS prevalence was 0.47% (95% CI = [0.41%, 0.53%]) and for boys it was 1.95% (95% CI = [1.84%, 2.06%]). This gave an updated boys-to-girls ratio of 4.18 after data linkage, smaller than the ratio of 6:1 in the school data. Updated ASD prevalence was highest among individuals aged 6 to 8 years and starting primary school at 1.54% (95% CI = [1.41%, 1.68%]) and decreased with age (Table 3).

**Table 3.** Adjusted prevalence and adjusted updated prevalence of ASD by health service in Chile.^a^

Health service	Adjusted school prevalence (95% CI)	Adjusted Bayesian clinical prevalence (Maximal 95% CI)
Aconcagua	0.43 [0.37, 0.50]	1.28 [1.21, 1.34]
Aisén	0.75 [0.63, 0.90]	1.60 [1.47, 1.73]
Antofagasta	0.83 [0.77, 0.88]	1.67 [1.61, 1.74]
Araucanía Norte	0.30 [0.24, 0.38]	1.15 [1.08, 1.21]
Araucanía Sur	0.37 [0.34, 0.41]	1.22 [1.16, 1.28]
Arauco	0.72 [0.62, 0.82]	1.56 [1.46, 1.66]
Arica	0.61 [0.54, 0.70]	1.46 [1.38, 1.54]
Atacama	0.31 [0.27, 0.37]	1.16 [1.10, 1.22]
Biobío	0.42 [0.37, 0.47]	1.27 [1.20, 1.33]
Chiloé	0.43 [0.36, 0.52]	1.28 [1.20, 1.36]
Concepción	0.77 [0.72, 0.83]	1.62 [1.56, 1.68]
Coquimbo	0.40 [0.36, 0.43]	1.24 [1.18, 1.31]
Iquique	0.43 [0.38, 0.49]	1.28 [1.22, 1.34]
Magallanes	0.83 [0.72, 0.96]	1.68 [1.56, 1.80]
Maule	0.30 [0.28, 0.33]	1.15 [1.09, 1.21]
Metropolitano Central	0.42 [0.38, 0.46]	1.26 [1.20, 1.33]
Metropolitano Norte	0.29 [0.26, 0.31]	1.13 [1.07, 1.20]
Metropolitano Occidente	0.34 [0.32, 0.36]	1.19 [1.12, 1.25]
Metropolitano Oriente	0.30 [0.27, 0.33]	1.15 [1.08, 1.21]
Metropolitano Sur	0.40 [0.37, 0.43]	1.25 [1.18, 1.31]
Metropolitano Sur Oriente	0.36 [0.34, 0.39]	1.21 [1.15, 1.27]
O’Higgins	0.42 [0.39, 0.46]	1.27 [1.21, 1.34]
Osorno	0.43 [0.37, 0.51]	1.28 [1.21, 1.35]
Reloncaví	0.42 [0.37, 0.47]	1.26 [1.20, 1.33]
Talcahuano	0.81 [0.74, 0.90]	1.66 [1.58, 1.74]
Valdivia	0.30 [0.26, 0.35]	1.15 [1.08, 1.21]
Valparaíso	0.68 [0.62, 0.74]	1.52 [1.46, 1.59]
Viña del Mar	0.66 [0.62, 0.70]	1.51 [1.44, 1.57]
Ñuble	1.29 [1.21, 1.37]	2.13 [2.05, 2.21]
Age band	Crude prevalence SSAS (95% CI)	Adjusted prevalence SSAS (95% CI)
6−8	1.54 [1.40, 1.67]	1.54 [1.41, 1.68]
9−11	1.34 [1.21, 1.46]	1.33 [1.21, 1.46]
12−14	1.08 [0.97, 1.19]	1.08 [0.97, 1.20]
15−18	0.96 [0.86, 1.07]	0.98 [0.87, 1.11]
Ethnicity	Adjusted prevalence School registry (95% CI)	Adjusted Bayesian prevalence (95% CI)
Mapuche	0.35% (0.32%−0.38%)	1.19 [0.37, 1.17]
Aymara	0.65% (95% CI = [0.55%, 0.80%])	1.50 [0.68, 1.48]
Other indigenous group	0.47% (95% CI = [0.39%, 0.63%])	1.34 [0.51, 1.31]
No indigenous groups declared	0.47% (95% CI = [0.46%, 0.48%])	1.32 [0.49, 1.30]

a Adjusted prevalence is from school data only. Adjusted updated prevalence is from linkage of school data and patient data. Prevalence for Servicio de Salud Araucanía Sur (SSAS) was calculated directly from linkage results. Prevalence for other health services was calculated by adding the adjusted prevalence delta to each health service’s adjusted prevalence from the school data only. Adjusted prevalence has 95% gamma confidence intervals. The width of the adjusted updated prevalence confidence intervals is the maximum of the school data−adjusted prevalence confidence intervals for each health service, and the adjusted prevalence delta confidence interval, except for SSAS which has the 95% gamma confidence intervals found earlier.

### Bayesian prevalence analysis

**Figure 3.**
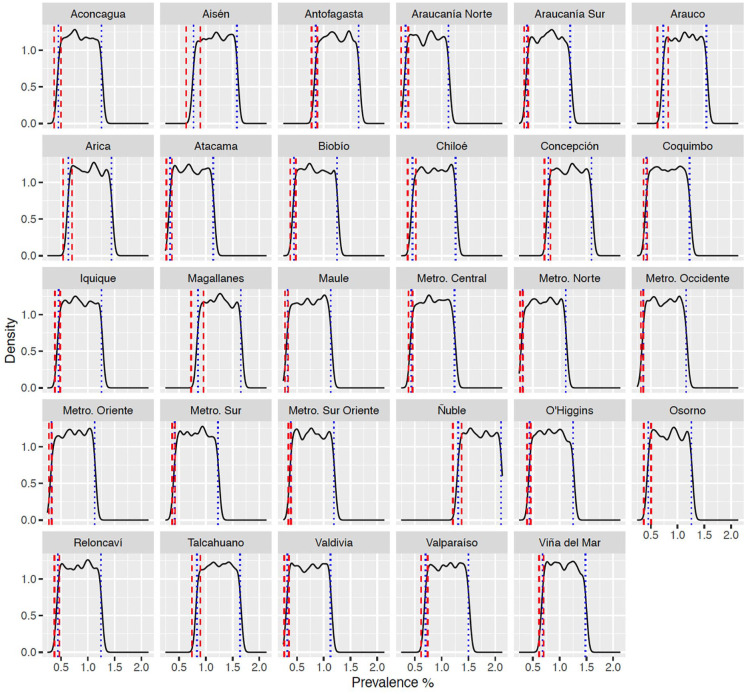
Posterior predictive distribution for health service−specific uniform priors. Posterior predictive distributions for ASD prevalence using adjusted case counts from the school data with a random effect on student’s health service. Modelling used uniform priors bounded below by health service−specific adjusted ASD prevalence from school data, and bounded above by health service−specific adjusted updated ASD prevalence from data linkage. Red dashed lines show the adjusted sample prevalence 95% gamma confidence intervals and blue dotted lines show the posterior 95% credible interval.

Bayesian clinical prevalence projections by health service with school-level ASD prevalence priors are shown in Figure 3. The posterior prevalence peaks can be considered lower bounds for the true ASD prevalence in each health service as they are based on children diagnosed with ASD receiving SEN support in Chilean schools. Bayesian prevalence projections were pulled upwards towards their priors by our approximate delta of unmet need (a). The posterior distributions were reflective of their uniform priors with posterior credible intervals slightly within the prior bounds (Table 3). Adding the adjusted prevalence delta to the national adjusted ASD prevalence found from the school data gave national adjusted updated ASD prevalence of 1.31% (95% Credible Interval [CrI] = 1.25%−1.38%) (Figure 3). This in turn gave a posited 40,113 children aged 6 to 18 years with ASD in Chile and corresponds to an estimated unmet need of 25,903 children who did not access SEED for ASD (posited count and unmet need are age- and sex-adjusted). The national ASD projected Bayesian prevalence for boys was 2.10% and for girls 0.50%, with a prevalence at age 6 to 8 of 2.30%, and in children who were 9 to 11 years of 2.05%, 12 to 14 years 2.11% and, finally, 15 to 18 years 1.91% (Table 3). In rural students, Bayesian prevalence was 1.42%, while for urban students this was 1.30%. National prevalence projections for Mapuche students was 1.19%, for Aymara 1.50%, for no declared Indigenous group 1.32%, and for other Indigenous groups was 1.34% and, finally, national prevalence projections was 1.03% for immigrants (Figure 3).

## Discussion

The field of ASD epidemiology has witnessed significant advancements in recent years, contributing to a deeper understanding of the prevalence, characteristics and impact of ASD within populations. One notable approach to enhance the accuracy and comprehensiveness of epidemiological data is linkage between school registries and clinical records. To the best of our knowledge, this is the largest ASD prevalence study in Latin America and the Caribbean to date, and one of the largest linking school registry and clinical records.

This study found an adjusted prevalence rate of 0.46% using school registry data and 1.22% in the electronic health records data from the SSAS region, with considerable gender disparity, showing a male-to-female ratio of 6:1. This ratio is considerably higher than the internationally recognized ratio of 4:1 (Roman-Urrestarazu et al., 2021, 2022) This pattern may reflect diagnostic and awareness biases towards males in school settings, as well as other systemic biases within health services, which can reduce the likelihood of autistic girls receiving a diagnosis. In Chile, there is currently no published evidence documenting such gender disparities, either for ASD or for the other neurodevelopmental conditions recognized under SEN services. This highlights a critical gap and underscores the need for future research and intervention development to address potential underdiagnosis and support equity in access to diagnosis and services. When reinvestigating the national-level prevalence estimates using Bayesian prevalence estimation, we estimated an updated national ASD prevalence in Chile of 1.31% (95% CrI = 1.25%−1.38%). This also showed that only 488 children (30.12%) accessed SEN support for ASD in the PIE programme in SSAS and that as many as 25,903 out of 40,113 (64.57%) autistic school-age ASD children in Chile do not access PIE for ASD, with boys being 50% more represented in the SEN programme, which further underscores the need for research into the factors driving these variations. This unmet need, although considerable, includes students with an ASD diagnosis at non-subsidized private schools that were not eligible for SEED but may have received other school-based support, students who received SEED for another condition and students who did not need or did not want school-based interventions. According to the Chilean disability service, 7.4% of adults with disabilities did not receive any formal education in their childhood, while in the rest of the population this percentage is limited to 1.3% (Ramos, 2015). This emphasizes the importance of optimizing resources to bridge the gap between diagnosed cases and access to appropriate interventions.

The study uncovered varying ASD prevalence rates among different ethnic groups. The Mapuche group had an adjusted school prevalence of 0.35% and a national projection based on the delta based on the same prior and assumptions obtained from the SSAS as 1.19%, while the Aymara group had a prevalence of 0.64% and a national projection of 1.50%. Immigrant children reported an adjusted prevalence of 0.19% and a projection of 1.03%. Our results are aligned with similar estimates from the United States and Europe and other high income countries (HIC) that describe a prevalence between 1% and 2% of children (Baron-Cohen et al., 2009; Diallo et al., 2018; Elsabbagh et al., 2012; Jariwala-Parikh et al., 2019; Maenner et al., 2014; Neik et al., 2014; Zeidan et al., 2022). The variation observed in prevalence across Chilean health services is probably the result of disparities in the professional services available. Chile is a country with an unequal distribution of medical and professional services. Central regions of the country, where almost 70% of the population lives, have around 85% of the country’s specialists (MINSAL, 2023). As a result, central regions of Chile have rates of specialists per 1000 inhabitants that are practically double those of the extreme regions, where ethnic groups such as the Mapuche mainly reside. The lower availability of diagnostic services may be involved in the observed disparities and shows the importance of addressing these inequities.

Using clinical records to validate epidemiological data coming from school registries offers several advantages that improve the accuracy of ASD prevalence from school registries done by linking across a wide range of sociodemographic variables, enabling researchers to perform population-level analyses with incomplete information, helping to identify potential disparities in access to services and resources. From a methodological viewpoint, our study adds to an emerging body of literature that leverages the ability to link large administrative and clinical data to model more accurate burden of disease estimates using Bayesian methods (Downing et al., 2023; Ince et al., 2021; Liseo & Tancredi, 2013), which is particularly suitable to model more accurate burdens of disease with fragmented health information systems (Downing et al., 2023). That said, some limitations need to be considered as well. The national projections of our Bayesian prevalence estimation are based on the assumption that the discrepancy between school and clinical records is uniform across all regions in Chile. While this is a valid starting point for the purpose of this methodology, we encourage follow-up analyses to be performed in which the discrepancies between school and clinical data can be examined in greater detail to further refine ASD prevalence estimates. While our data capture the vast majority of Chilean pupils aged 6 to 18 years, our findings may not be directly translatable to other countries in the Latin American region given the heterogeneity in health and education systems, as well as possible differences in the make-up of health inequalities (Roman-Urrestarazu & van Kessel, 2024). Nevertheless, our findings remain particularly relevant for the purpose of health system planning in Chile, especially in light of the 2023 ASD law.

To the best of our knowledge, this is the first population-based assessment of ASD prevalence in Chile that takes advantage of linking clinical and school registry data to produce ASD prevalence rates. Moving forward, further research should delve into areas where disparities in SEN educational services might exist, particularly including insurance status as this is a variable crucial in measuring access to services and that in countries like the United States, has not been assessed its impacts to ASD prevalence. Efforts to explore the relationship between ASD prevalence, sex and unmet need for SEN should continue, recognizing the complexities involved in access to diagnostic services, and regional variations of health and educational access in Latin America and the Caribbean.

## Supplemental Material

sj-docx-1-aut-10.1177_13623613251342310 – Supplemental material for Bayesian prevalence of autism and unmet special education needs in Chile in a sample of three million school-age children Supplemental material, sj-docx-1-aut-10.1177_13623613251342310 for Bayesian prevalence of autism and unmet special education needs in Chile in a sample of three million school-age children by Andres Roman-Urrestarazu, Adele Tyson, Gabriel Gatica-Bahamonde, Robin van Kessel, Justin Yang, Carola Mansilla, Isabel Zuniga, Alejandra Méndez-Fadol, Blanca Larrain, Ricardo Garcia, Damaris Koch, Tamsin Ford, Wim Groot, Milena Pavlova and Katarzyna Czabanowska in Autism
