# Supplementary material for: Bayesian prevalence of autism and unmet special education needs in Chile in a sample of three million school-age children
Source: Autism. 2025 Aug 4;29(10):2501–12. doi: 10.1177/13623613251342310 (PMC12417606; doi:10.1177/13623613251342310)
Supplement: sj-docx-1-aut-10.1177_13623613251342310 – Supplemental material for Bayesian prevalence of autism and unmet special education needs in Chile in a sample of three million school-age children [file sj-docx-1-aut-10.1177_13623613251342310.docx]

Supplementary materials

Table of Contents

[eMethods 1. 2](#_Toc195702514)

[eMethods 2. Supplementary information on the small clinical dataset. 2](#_Toc195702515)

[eMethods 3. 3](#_Toc195702516)

[eMethods 4. Probabilistic record linkage. 3](#_Toc195702517)

[eMethods 5. 5](#_Toc195702518)

[eMethods 6. 6](#_Toc195702519)

[eTable 1. Crude and adjusted prevalence rate per health service region using school data. 11](#_Toc195702520)

# eMethods 1.

Chile’s smallest administrative subdivision is called a commune. Chile has 346 communes are grouped into 56 provinces, which are themselves grouped into 16 regions. Two issues due to boundary changes were corrected. Firstly, the municipality now called Tocopilla, which falls across the Antofagasta and Tarapaca regions was formerly two municipalities, namely Tocopilla in Antofagasta and Pozo Almonte in Tarapaca. The old names have been retained to ensure appropriate region mapping and they are mapped to the Antofagasta and Iquique health services respectively. Secondly, the municipalities recorded as belonging to the former Nuble sub-region of the Bio-Bio region were mapped to their corresponding municipalities in the recently formed Nuble region

# eMethods 2. Supplementary information on the small clinical dataset.

The small clinical dataset is understood to include every such patient resident in the municipalities of Curarrehue, Loncoche, Pucón and Villarica in the SSAS catchment of Chile’s Araucanía region, and include some such patients resident in the municipalities of Cunco, Freire, Gorbea, Nueva Imperial, Pitrufquén, Temuco, Teodoro Schmidt and Toltén in the SSAS catchment. It also includes some such patients resident in the municipalitys of Algarrobo (Valparaíso San Antonio health service catchment, Valparaíso region), Cabo de Hornos (Magallanes health service catchment, Magallanes y Antártica Chilena region), Diego de Almagro (Atacama health service catchment, Atacama region), Hijuelas (Viña del Mar Quillota health service catchment, Valparaíso region), Machalí (Libertador B.O’Higgins health service catchment, Libertador B.O’Higgins region), Panguipulli (Valdivia health service catchment, Los Ríos region), Pencahue (Maule health service catchment, Maule region), Pica (Iquique health service catchment, Tarapacá region), Quinta Normal (Metropolitano Occidente health service catchment, Metropolitana de Santiago region) and Tocopilla (Antofagasta health service catchment, Antofagasta region).

# eMethods 3.

Kolmogorov-Smirnov permutation tests found no significant difference in frequency of sexes between matched (12·88% female) and unmatched SSAS school records (12·16% female). We found a significant difference (p<0.001) in the frequency of sexes between matched (12·88% female) and unmatched patient records (21·52% female). This difference was likely due to the male to female ratios differing across the datasets: the SSAS school data of students with autism was 12·47% female, the 49 patient data was 20·06% female and the matches were 12·50% females. Permutation testing found that for the SSAS school data, matched (39·91% resident in Temuco) and unmatched records (56·08% resident in Temuco) differed significantly by commune, and that there was no significant difference in the patient data by commune between matched (40·34% resident in Temuco) and unmatched records (40·16% resident in Temuco). This appeared to be driven by the matchability of students and patients living in Temuco, the most prevalent commune. For the SSAS school data by proxy SES there was a (p<0.001) significant difference between matched (90·56% status of 1, 5·58% status of 2, 0·00% status of 3 and 3·86% with unknown status) and unmatched (80·78% status of 1, 13·73% status of 2, 0·39% status of 3 and 5·10% with unknown status) records. For the patient data there was a somewhat significant difference between matched (38·2% status of 1, 58·37% status of 2, and 3·43% status of 3) and unmatched (42·69% status of 1, 55·64% status of 2 and 1·66% status of 3) records. Again this likely reflected different frequencies of the SES values across datasets. Kolmogorov-Smirnov permutation testing was not conducted for date of birth as this feature contained too many categories for results to be meaningful.

# eMethods 4. Probabilistic record linkage.

For data linkage, the School Registry data was restricted to students with autism that were living in municipalitys in the SSAS catchment in 2021 to maximise comparability with the clinical data. One false empty record was added to the school dataset before linkage that allowed the algorithm to correctly match on SES. This false record was only used during linkage, did not match to any patient records, and was removed before comparing matched and unmatched records. This was restricted to appointments for individuals resident in municipalities in the SSAS catchment as the data is believed to be complete for this catchment area only. It was also restricted to patients aged 6-18 as of 30th June 2021 to maximise compatibility to the school data. Appointment year was not restricted in order to retain more data and thus maximise linkage opportunities, and only patients of female and male sex were present.

Sex, date of birth, municipality of residence, autism diagnosis, and the proxies for socio-economic status (i.e., monthly school fees in the school data and mode health insurance contributions in the patient data) were available in both school and clinical datasets for matching. Although only students diagnosed with autism are included in the school dataset, the clinical dataset comprises primarily patients diagnosed with autism, some patients diagnosed with an intellectual disability, and some diagnosed with both. The autism diagnosis feature is therefore included in the features to match on to encourage matching of school records to clinical records for patients with an autism diagnosis, as well as to allow for matching to clinical records for patients with only a diagnosis of intellectual disability when no suitable patient with autism is present.

All possible pairs of blocked matches were generated and agreement weights were calculated for each feature using expectation maximisation. These feature weights were then aggregated into a weight for the pair. Observations with missing values were retained as this linkage method is robust to missingness. As similarity comparison method, we used exact matching for municipality of residence, autism diagnosis and socio-economic status. There was no value in using a string comparison method for municipality of residence as all municipality names were already standardised and two municipalities having similarly spelled names does not increase the likelihood of a match between those municipalities.

To link the datasets, we applied a comparator cut-off value of 0·99 for municipality of residence and autism diagnosis as these are expected to be fairly accurate features, and a cut-off of 0·60 was supplied for socio-economic status as it is a loosely defined proxy. These values was chosen iteratively through trial-and-error to ensure the algorithm prioritised matching on diagnosis of autism above matching on socio-economic status. Linkage was implemented using R’s RecordLinkage package. This included consideration of the average frequencies of categories in each feature and estimated errors rates were supplied: the default estimated error rate of 0·01 was supplied for the commune of residence and diagnosis with autism features as they were expected to be fairly accurate features, and an estimated error rate of 0·1 was supplied for the socio-economic status feature to reflect that it was a loosely defined proxy. Pairs were then selected based on weight to create a 1-1, bipartite, matching between school records and patients. These matches were examined to ensure a patient that lived in multiple municipalities had matched to only one school record and to assess the plausibility of matches made to patients diagnosed with intellectual disability instead of autism. Clinical data was subsequently de-duplicated to a single row per patient. In case a patient had multiple entries, the municipality of their matched record was chosen. The school data for students aged 6-18 in the SSAS catchment area and the deduplicated patient-level data for patients aged 6-18 in SSAS were combined to form the linked dataset, leaving out the deduplicated patient records that were a match to student records to ensure those individuals were not present twice in the linked data.

For the school and clinical datasets, each record was classified as either matched or unmatched based on whether it appeared in the bipartite matching. The discrete Kolmogorov-Smirnov test was used to compare matched and unmatched records within each dataset for each of the features used for matching, excluding date of birth as it has too many categories to have meaningful results and autism diagnosis as it is uniformly true in the school dataset and therefore not informative. Missing values in the socio-economic status feature were omitted before testing. Permutation tests were then performed for each of the features tested in each dataset by permuting the matched status 2000 times and recomputing the discrete Kolmogorov-Smirnov test for each permutation. The p-values for the Kolmogorov-Smirnov tests on the observed data were then compared to the distributions of p-values for the permuted data to determine the significance of the observed results. For each patient that had lived in more than one commune and therefore appeared more than once in the patient data, only one match to an SSAS school record was made, meaning the matching was bijective for SSAS school records and unique patients.

# eMethods 5.

In our clinical validation dataset, we also used a proxy for socioeconomic disadvantage based on health service users’ social health insurance status.^5,12^ Membership to the public insurance scheme has been used extensively as a proxy for income since it is linked to wage-deducted contributions, with privately insured users preferring hospitals in the private network and rarely attending public clinics.^12^ We further used students’ school fee status as a proxy for socio-economic status. Students with free schooling were assigned low SES as families with low SES are entitled to schooling rebates. Students paying $1,000-$100,000 monthly were given medium SES, and students paying more than $100,000 monthly were assigned high SES. Students’ ethnicity was mapped to being a member of the Mapuche Indigenous group, being a member of another Chilean Indigenous group, or not being a member of an Indigenous group based on their recorded ethnicity, which can take at most one value. Students with ethnicity recorded as ‘no registry’ were mapped to not being a member of an Indigenous group.

# eMethods 6.

We used the following Bayesian model:

(1) y_i_|(n_i_, θ_i_) ∼ Binomial(n_i_, θ_i_)

in which y_i_ refers to the adjusted count of ASD cases in health service i, n_i_ represents the number of students in health service i, and θ_i_ designates the prevalence of ASD in health service i. This model is paired with a prior distribution of θ_i_ that follows:

(2) θ_i_ ∼ Beta(a, b)

in which a captures a prior of the ASD prevalence rate and b represents the corresponding prior of the standard deviation of the ASD prevalence rate. When combined, the subsequent posterior distribution can be derived to model the ASD prevalence rate in health service i given the number of children diagnosed with ASD in health service i and the total population of health service i:

(3) θ_i_|(y_i_, n_i_) ∼ Beta(y_i_ + a, n_i_ − y_i_ + b)

Four priors for θ_i_ were used when fitting the Bayesian prevalence model to account for data gaps.

Prior 1 constituted a conjugate beta prior common to all health services, constructed with the national adjusted ASD prevalence from the frequentist analysis of the school registry and its standard deviation as the mean and standard deviation of the prior. This prior was suitable seeing as this adjusted prevalence in the school data provides a plausible lower bound on the prevalence of ASD in Chile.

Prior 2 involved a conjugate beta prior specific to each health service, developed using the health service-specific adjusted ASD prevalence estimates from the school registry and their respective prior means and standard deviations. This prior was also suitable because it was extending the previous prior to each of the random effect categories and reflected students receiving SEN. On its own, this prior is expected to give uninformative posteriors because it effectively duplicated the information in the sample data. However, it was suitable as a more specific lower bound on the plausible prevalence of ASD in each health service.

Prior 3 was a conjugate beta prior specific to each health service, derived using the adjusted prevalence projected from the linked data from SSAS and their standard deviations from their maximal 95% confidence intervals as the prior means and prior standard deviations respectively. This prior was suitable as captures the clinical information provided by the linkage and includes all pupils with clinical diagnoses and with ASD SEN from the school registry, additionally it has narrow standard deviations which will model a theoretical upper bound on the prevalence of ASD in each health service.

Prior 4 comprised a uniform prior specific to each health service using the adjusted ASD prevalences from the school data for each health service as its lower bounds, and the projected prevalences ratio from the linked data for each health service as its upper bounds. This prior is suitable because it captures the information from both the school and linked datasets, without specifying where within these bounds the true prevalences are likely to be.

**eMethods 7**

**Registration process for PIE:**

The School Integration Programme (Programa de Integración Escolar, PIE) is an inclusive strategy within the school system, the purpose of which is to provide resources and tools to contribute to the continuous improvement of the quality of education, favouring access, participation and progress in the learning of students, especially those with special educational needs (NEE).

The objective of the PIE is to provide a pertinent, relevant and meaningful education that takes into account the diversity of our students in the teaching-learning process, through the strengthening of pedagogical practices and the provision of support inside and outside the classroom, by a multidisciplinary team specializing in educational needs.

1. **Operation**

The PIE liaises with all the different sectors of the educational community (pupils, teachers, parents, management, guidance, school coexistence and inspectorates), focusing mainly on the collaborative work of the classroom team made up of the subject teacher, the special needs teacher and specialist professionals if required, who directly support the academic process of our students, through the implementation of pedagogical strategies that allow access to and progress within the curriculum, considering the diversity of learning styles and paces present in a course group.

Students receive support from the special needs teacher in the classroom, especially in language and mathematics, at least three times a week. In addition, specialists in the fields of psychology, speech therapy and occupational therapy are included for those students with a permanent special educational need.

Currently, there are no maximum quotas per year group. The PIE must attend to all children with SEN.

1. **Definitions**

Transient Special Educational Needs: (Spanish acronym: NEET).

These are needs that arise during a certain period of schooling and that can diminish and even disappear with a rigorous work plan coordinated between the family, the school and the specialists involved.

Within the NEET, we can find: Attention Deficit Disorder with or without Hyperactivity (ADD/ADHD); Specific Learning Difficulties (SpLD); Borderline Intellectual Functioning.

Permanent Special Educational Needs: (Spanish acronym: NEEP).

These are needs that a person presents throughout their life, and therefore throughout their school period. These needs can be addressed by reducing barriers present within the establishment, which facilitates their integration into the school dynamic, as long as the required support is provided by the family, school and specialists involved in each case.

Among the NEETs, we can find: Autism Spectrum Disorder (ASD); Sensory Disabilities (hearing or visual); Intellectual Disability (ID).

1. **PIE Admission Procedure**

First stage: students who require specialized support can be referred through the following channels:

- The family must inform the school if the student has been diagnosed by a specialist (neurologist, psychiatrist, speech therapist, psychologist, educational psychologist).
- Teachers must report if there is a student who needs to be evaluated.

Second stage: it is decided whether the student enters the School Integration Programme, through the following process:

1. The family must present a certificate with an up-to-date diagnosis, issued by a specialist.
2. The family must meet with PIE professionals to complete an interview (anamnesis) that provides evidence of the student's evolutionary, medical, social and emotional development history.
3. The family must sign an authorization for the PIE team to evaluate the student's pedagogical and social performance.
4. After reviewing the background information gathered, a psycho-pedagogical and/or speech therapy and/or occupational and/or psychological evaluation is also carried out, as appropriate.
5. A general health evaluation is carried out by a pediatrician or family doctor, and a specialized evaluation is carried out by a child neurologist or child psychiatrist.

Third Stage: the continuation or graduation of each student belonging to the PIE is reconsidered annually, with the team's decision being justified by means of records that show a reassessment process carried out at the end of the school year.

# eTable 1. Crude and adjusted prevalence rate per health service region using school data.

| **Health service** | **Crude prevalence (95% CI)** | **Adjusted prevalence (95% CI)** |
| --- | --- | --- |
| Aconcagua | 0·44 (0·38, 0·50) | 0·43 (0·37, 0·50) |
| Aisén | 0·75 (0·63, 0·87) | 0·75 (0·63, 0·90) |
| Antofagasta | 0·84 (0·79, 0·89) | 0·83 (0·77, 0·88) |
| Araucanía Norte | 0·30 (0·24, 0·36) | 0·30 (0·24, 0·38) |
| Araucanía Sur | 0·37 (0·34, 0·40) | 0·37 (0·34, 0·41) |
| Arauco | 0·73 (0·64, 0·83) | 0·72 (0·62, 0·82) |
| Arica | 0·61 (0·54, 0·68) | 0·61 (0·54, 0·70) |
| Atacama | 0·31 (0·26, 0·35) | 0·31 (0·27, 0·37) |
| Biobío | 0·43 (0·38, 0·48) | 0·42 (0·37, 0·47) |
| Chiloé | 0·45 (0·38, 0·52) | 0·43 (0·36, 0·52) |
| Concepción | 0·78 (0·73, 0·84) | 0·77 (0·72, 0·83) |
| Coquimbo | 0·41 (0·38, 0·45) | 0·40 (0·36, 0·43) |
| Libertador B.O'Higgens | 0·43 (0·40, 0·47) | 0·42 (0·39, 0·46) |
| Maule | 0·31 (0·28, 0·33) | 0·30 (0·28, 0·33) |
| Reloncaví | 0·42 (0·38, 0·47) | 0·42 (0·37, 0·47) |
| Iquique | 0·45 (0·40, 0·50) | 0·43 (0·38, 0·49) |
| Magallanes | 0·83 (0·72, 0·94) | 0·83 (0·72, 0·96) |
| Metropolitano Central | 0·42 (0·38, 0·46) | 0·42 (0·38, 0·46) |
| Metropolitano Norte | 0·29 (0·27, 0·32) | 0·29 (0·26, 0·31) |
| Metropolitano Occidente | 0·36 (0·34, 0·38) | 0·34 (0·32, 0·36) |
| Metropolitano Oriente | 0·30 (0·28, 0·33) | 0·30 (0·27, 0·33) |
| Metropolitano Sur | 0·41 (0·39, 0·44) | 0·40 (0·37, 0·43) |
| Metropolitano Sur Oriente | 0·37 (0·34, 0·39) | 0·36 (0·34, 0·39) |
| Osorno | 0·44 (0·38, 0·51) | 0·43 (0·37, 0·51) |
| Talcahuano | 0·84 (0·76, 0·92) | 0·81 (0·74, 0·90) |
| Valdivia | 0·31 (0·27, 0·35) | 0·30 (0·26, 0·35) |
| Valparaíso San Antonio | 0·69 (0·64, 0·75) | 0·68 (0·62, 0·75) |
| Viña del Mar Quillota | 0·67 (0·63, 0·71) | 0·66 (0·62, 0·70) |
| Ñuble | 1·32 (1·24, 1·40) | 1·29 (1·21, 1·37) |
